# A combination of climatic conditions determines major within-season dengue outbreaks in Guangdong Province, China

**DOI:** 10.1186/s13071-019-3295-0

**Published:** 2019-01-21

**Authors:** Xia Wang, Sanyi Tang, Jianhong Wu, Yanni Xiao, Robert A. Cheke

**Affiliations:** 1 School of Mathematics and Information Science, Shaanxi Normal University, Xi’an, 710119 People’s Republic of China; 2 Centre for Disease Modelling, York Institute for Health Research, York University, Toronto, ON M3J 1P3 Canada; 3 School of Mathematics and Statistics, Xi’an Jiaotong University, Xi’an, 710049 People’s Republic of China; 4 Natural Resources Institute, University of Greenwich at Medway, Central Avenue, Chatham Maritime, Kent, ME4 4TB UK; 5 Department of Infectious Disease Epidemiology, School of Public Health, Faculty of Medicine (St Mary’s campus), Imperial College London, Norfolk Place, London, W2 1PG UK

**Keywords:** Dengue, *Aedes*, Precipitation, Temperature, Multi-scale model, Short-term forecast, Effective reproduction number

## Abstract

**Background:**

China’s Guangdong Province experienced a major dengue outbreak in 2014. Here we investigate if the weather conditions contributing to the outbreak can be elucidated by multi-scale models.

**Methods:**

A multi-scale modelling framework, parameterized by available weather, vector and human case data, was used to examine the integrative effect of temperature and precipitation variation on the effective reproduction number (ERN) of dengue fever.

**Results:**

With temperature in the range of 25–30 °C, increasing precipitation leads to an increase in the ERN with an average lag of 10 days. With monthly precipitation fixed, the more regular the pattern of rainfall (i.e. higher numbers of rainy days), the larger is the total number of adult mosquitoes. A rainfall distribution peaking in June and July produces a large ERN, beneficial to transmission. Climate conditions conducive to major outbreaks within a season are a combination of relatively high temperature, high precipitation peaking in June and July, and uninterrupted drizzle or regular rainfall.

**Conclusions:**

Evaluating a set of weather conditions favourable to a future major dengue outbreak requires near-future prediction of temperature variation, total rainfall and its peaking times. Such information permits seasonal rapid response management decisions due to the lags between the precipitation events and the realisation of the ERN.

**Electronic supplementary material:**

The online version of this article (10.1186/s13071-019-3295-0) contains supplementary material, which is available to authorized users.

## Background

Dengue fever, that can cause severe influenza-like illness and potentially lethal complications, has been spreading extensively in tropical and subtropical regions. It is the fastest spreading emerging infectious disease in the Asia Pacific region, where it was first recognized in the 1950s in the Philippines and Thailand, and there has been a persistently increasing trend with multi-year oscillations in disease occurrences in the Western Pacific Region. In China, where dengue fever was first imported from Southeast Asia in 1917, few outbreaks were reported until a sudden major outbreak in Foshan city in Guangdong Province in 1978 [1, 2]. Since then outbreaks have been occurring almost annually in southern China in general and in Guangdong Province in particular, with *Aedes albopictus* being the dominant vector [3]. The 2014 outbreak in Guangdong Province was the most serious one in mainland China to date and here we identify its key causative factors.

Dengue epidemics are seasonal, given the life-cycles of its vectors (*Ae. aegypti* and *Ae. albopictus*) whose reproduction, development and mortality rates are regulated by temperature, precipitation and humidity [4–7]. Correlations between weather factors and dengue outbreaks [8–10] have been incorporated into mathematical models of the dynamics of both mosquito populations and dengue fever disease transmission [11–14].

In particular, recent studies [15–18] have considered weather, disease importation, spatio-temporal patterns of disease spread and control measures, all possible contributors to the 2014 Guangzhou outbreak in Guangdong Province. Li et al. [19] suggested that the main reasons for the outbreak were late control measure implementation, numerous imported cases from the end of April to early July, and high precipitation from May to August. In 2016 Cheng et al. [15] showed that early case importations and excessive rainfall were the most important determinants but in 2017, they [16] concluded that the high number of imported cases in May and June rather than the weather conditions was primarily responsible for the early outbreak. Using a generalized additive model to examine dengue fever transmission in Guangzhou from 2005 to 2015, Xu et al. [18] showed that effects of rainfall and temperature on mosquito abundance and dengue transmission are key to explaining the temporal dynamics of dengue fever incidence. Additional questions, however, remain in terms of quantifying and evaluating the impact of weather factors on dengue fever prevention and control: how do temporal variations in the distribution of the rainfall, given a fixed total amount of precipitation in a given period, affect an outbreak? Does low rainfall invariably lead to minor outbreaks? To what extent and how fast and reliably can these key factors be incorporated into models to predict the development of a potentially large outbreak within a season? How long is the delay between the onset of weather conditions favourable for disease spread and observations of disease incidence, and can this delay permit more effective prevention and control measures?

We address these questions using a set of mathematical modelling tools incorporating weather condition impacts on vector population dynamics, vector-human interaction patterns and human incidence generation. We first calibrated the model using the weather and human incidence data from 2012–2014 to identify key factors behind the major outbreak of 2014; we then validated and analysed it with date fitting to 2014–2017 events; and finally we used the model to quantify the weather conditions favourable for a large outbreak and illustrate the potential for providing early warning of the epidemic situation to inform interventions in real time.

## Methods

### Data

**Fig. 1:**
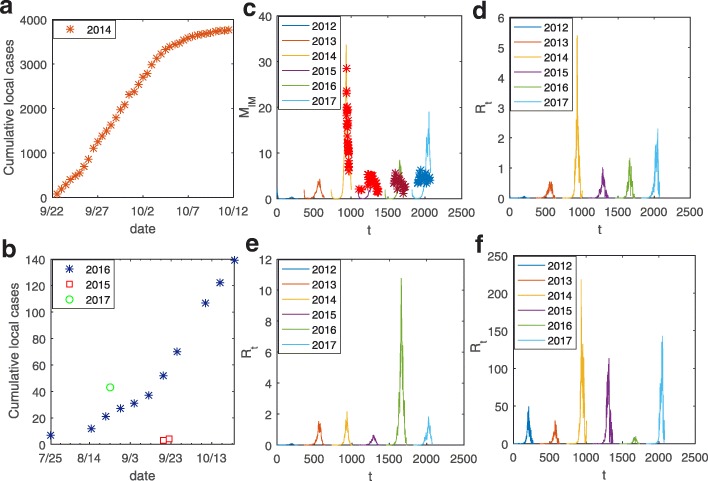
**a**, **b** The cumulative number of local cases. The X-axis represents the date. **c** Comparison of the BI surveillance data (stars) and the predicted numbers of immature mosquitoes (lines). **d** The effective reproduction number from 2012 to 2017. **e** The effective reproduction number from 2012 to 2017, but with the temperature of every year taken as the mean from 2012 to 2016. **f** The effective reproduction number from 2012 to 2017, but with the precipitation of every year taken as the mean from 2012 to 2016

Since 2002, the city of Guangzhou has been using the conventional surveillance method [20], the Breteau index (BI), the common index for *Aedes* density surveillance, that is calculated from the number of containers with mosquito eggs or larvae per 100 houses inspected. From September 22nd to October 30th 2014, Guangzhou Center for Disease Control and Prevention released the BI data almost daily, but in 2015, 2016 and 2017 it was also reported almost weekly, see Fig. 1c. For details of the weather and case data used (Fig. 1a, b), see Additional file 1: Text S1 and Table S1.

### Model equations

A stage-structured mosquito population model was formulated as follows [21]:

1 $$ \left\{\begin{array}{l}{M}_{IM}^{\prime }(t)=b(t){M}_A(t)-d(t){M}_{IM}(t)-{\mu}_1(t){M}_{IM}(t)\\ {}{M}_A^{\prime }(t)=d(t){M}_{IM}(t)-{\mu}_2(t){M}_A(t)\ \\ {}{W}^{\prime }(t)=\lambda (t)-\delta (t)W(t)\ \end{array}\right. $$

where ^'^ means the derivative with respect to *t*; *M*_*IM*_ and *M*_*A*_ are the amounts of immature and mature mosquitoes; *W*is the moisture index; *b*(*t*) is the egg-laying rate, which depends on the moisture index; *d*(*t*) is the development rate of the immature mosquitoes; *μ*_*i*_(*t*)(*i* = 1, 2) are the mosquito mortality rates; *λ*(*t*) is the precipitation; and *δ*(*t*) is the evaporation rate (as shown in Table 1). According to the relationship between the relative vectorial capacity and the reproduction number [22, 23], we obtained the expression of the effective reproduction number (ERN), which is the basic reproduction number of the mosquito without considering its variation with time *t*.

**Table 1:** Definitions of the parameters used in the model

Parameter	Definition (units)
*a*	The average biting rate (per day)
*b*_*m*_	Transmission probability from human to mosquito (per bite)
*b*_*h*_	Transmission probability from mosquito to human (per bite)
*n*	The duration of the extrinsic incubation period (days)
*T*_*h*_	The infectious period (days)
*M*_*H*_	The ratio between the mosquito and human populations
*b*(*t*)	The egg-laying rate (per mosquito per day)
*d*(*t*)	The development rate of immatures (per day)
*μ*_1_(*t*)	The daily mortality rate of immatures (per day)
*μ*_2_(*t*)	The daily mortality rate of adults (per day)
*λ*(*t*)	The total daily precipitation (mm)
*δ*(*t*)	The evaporation rate (mm)

2 $$ {\mathrm{R}}_{\mathrm{t}}={\mathrm{R}}_{\mathrm{VC}}\left(\mathrm{t}\right){\mathrm{T}}_{\mathrm{h}}{\mathrm{M}}_{\mathrm{H}}\left(\mathrm{t}\right) $$

where *T*_*h*_ is the infectious period, the ratio between the mosquito and human populations is *M*_*H*_(*t*) = *cM*_*A*_(*t*)/*N*_*h*_ with constant *c* and human population size *N*_*h*_. The vectorial capacity relative to the vector-to-human population ratio *R*_*VC*_(*t*) gives *R*_*VC*_(*t*) = *a*^2^(*t*)*b*_*h*_(*t*)*b*_*m*_(*t*) exp(−*μ*_2_(*t*)*n*(*t*))/*μ*_2_(*t*)*.* The function *a*(*t*) is the average daily vector biting rate, *b*_*h*_(*t*) represents the probability of vector to human transmission per bite, *b*_*m*_(*t*) is the probability of human to vector infection per bite, *n*(*t*) is the duration of the extrinsic incubation period (EIP), and *μ*_2_(*t*) is the adult vector mortality rate, which is the same as that in the mosquito model. These time dependent parameters are functions of temperature at time *t* (see Additional file 1: Text S1 and Table S2). The temperature at any time within a day was estimated using the sinusoidal hourly temperature variation between the maximum and minimum [13]. Detailed definitions for parameters and/or functions are given in Table 1.

### Parameter estimation

Parameters involved in the mosquito dynamics model were derived from a previous paper [21]. To estimate other unknown parameters in the transmission model, we assumed that the number of local incident cases follows a Poisson distribution, so that the likelihood function was obtained. The daily numbers of new cases in Guangzhou city during September 22nd to October 30th 2014 were used to estimate parameters by the Markov Chain Monte Carlo (MCMC) method and the generation interval-informed method [24]. For details see Additional file 1: Text S1 and Table S3.

## Results

### The effective reproduction number (ERN *R*_*t*_)

Incorporating the mosquito dynamics model into Eq. 2, we calculated the ERN as shown in Fig. 1d, which shows a clear seasonal pattern with the largest peak in 2014 (with a peak value above 5) and the smallest peak in 2012. The results agreed well with the actual epidemic size, shown in Additional file 1: Table S1. The ERN for 2014 showed a slowly increasing trend (Fig. 1d), given the relatively consistent control measures and/or climate in that year. The ERN in 2013 (Fig. 1a, b) was lower than in 2015, 2016 and 2017, but the total number of cases in 2014 was higher than in the other three years, possibly resulting from varying levels of control measure implementations before and after 2014.

### Effects of precipitation and temperature on the ERN

To investigate effects of precipitation and temperature on the abundance of mosquitoes and dengue fever transmission, we investigated how the mosquito population and the ERN vary if the yearly temperature or precipitation is substituted by the mean from 2012 to 2016. Thus, we examined the impact of temperature (or precipitation) by using a uniform precipitation (or temperature) throughout the study period. Figure 1e gives the simulation results when the yearly temperature was fixed as the mean temperature from 2012 to 2016, and shows that the ERNs in years 2013 and 2016 became larger than those in Fig. 1d, especially in 2016 for which it was the largest, indicating that the precipitation in 2016 contributed substantially to the mosquito reproduction. Fixing the yearly precipitation as the uniform mean precipitation from 2012 to 2016 revealed that the yearly ERN becomes larger than that with a non-uniform precipitation; particularly, the *R*_*t*_ in 2014 remained the largest (Fig. 1f). This indicates that the temperature in 2014 was the most beneficial for mosquito reproduction and dengue fever transmission. The ERN of 2016 was the smallest for the uniform precipitation (Fig. 1f), which means that the temperature in 2016 was the most disadvantageous for dengue transmission, which explains why the 2016 outbreak was not very big.

**Fig. 2:**
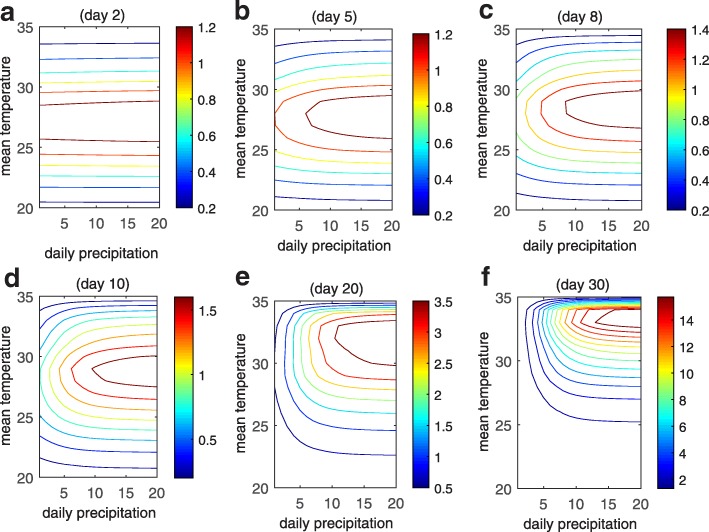
Contour plots of the effective reproduction number on days 2, 5, 8, 10, 20 and 30 (**a**-**e**)  when DTR = 5

To further analyze effects of temperature on dengue transmission, we produced a contour plot of the ERN with respect to the mean temperature and the daily precipitation (Fig. 2). The daily mean temperature and the daily temperature range (DTR) were used to calculate the temperature at any time and then to obtain these time dependent parameters. We assumed that it rains every day with the same precipitation and ran the model (1) with (2) for 30 days and obtained the ERN on days 2, 5, 8, 10, 20 and 30. The plots show that increasing the mean temperature has a considerable and immediate impact on the ERN. When the mean temperature is relatively low (below 25 °C) or very high (above 33 °C), increasing daily precipitation hardly affects the ERN (Fig. 2a, b), while for suitable temperatures (between 25–33 °C) varying daily precipitation greatly affects the ERN but a few days later (Fig. 2c-f), and hence there is a lag of a few days between the rainfall and an ERN increase. Hence the most beneficial temperature for transmission is around 30°C when DTR = 5, while very high or low temperatures are not conducive to transmission. Also, the optimal temperature that maximises the ERN becomes larger with time (Fig. 2c-f), agreeing with the fact that the optimal mosquito survival temperature is relatively small (around 27 °C; [21]) but the optimum for dengue fever transmission is relatively large (around 32 °C; [25]). This is because at the start of the simulation there are few mosquitoes and only a single infected individual, so temperature initially mainly affects the growth of mosquitoes, then affects both the mosquito reproduction and the biting rate and transmission probability per bite with an increasing number of mosquitoes. Further, comparing the values of *R*_*t*_ in Fig. 2 indicates that continuous rainfall for a few days may not lead to a noticeable increase in ERN *R*_*t*_, while continuous rainfall for more than 20 days will lead to a significant increase in ERN.

### Effects of monthly patterns of rainfall on the mosquito population and the ERN

**Fig. 3:**
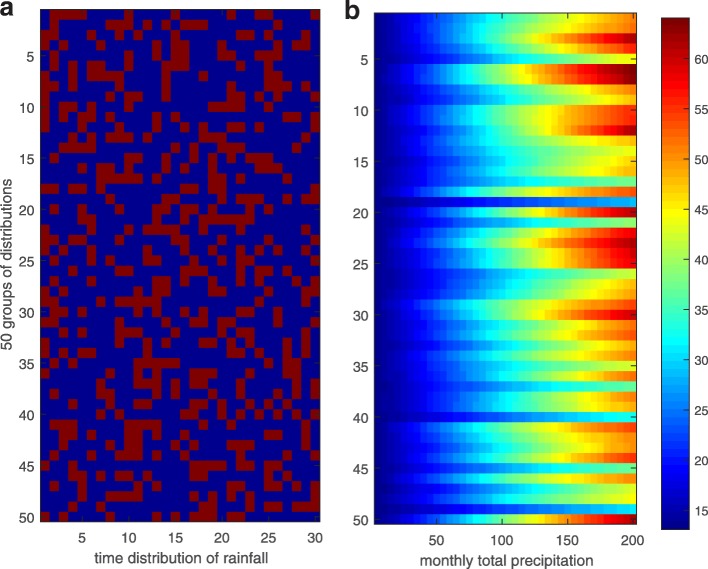
**a** The patterns of monthly rainfall. Dark blue represents no rain, the red represents rain. **b** The total amount of mosquitoes with respect to the total precipitation and the rainfall pattern

Figure 1e shows that the ERN in 2015 was still smaller than that in 2014 even with the uniform temperature, so the total precipitation in 2015 might have contributed less to the ERN than it did in 2014, although this was not borne out by the actual events. This implicitly means that the frequency and temporal distribution of the rainfall may be key factors that significantly affect the reproduction of the mosquito and hence the transmission of dengue fever. To examine the effect of precipitation on the mosquito population, we conducted numerical studies by fixing certain values of the daily mean temperature, the DTR and the total number of rainy days within one month and observing the values of *M*_*A*_ in one month. We used sampling without replacement to generate the date of rainfall from 1st to 30th of a month, and plotted the distributions in Fig. 3a. Figure 3b shows the total amount of adult mosquitoes within 30 days with respect to the total precipitation and its temporal distributions. It shows that the larger is the total precipitation the more the mosquitoes. Interestingly, different distributions of rainy days can cause very significant differences in mosquito abundance. In particular, when the total precipitation is 200 mm, the total amount of adult mosquitoes produced within 30 days can be larger than 60 or below 40 with different rainfall temporal distributions.

**Fig. 4:**
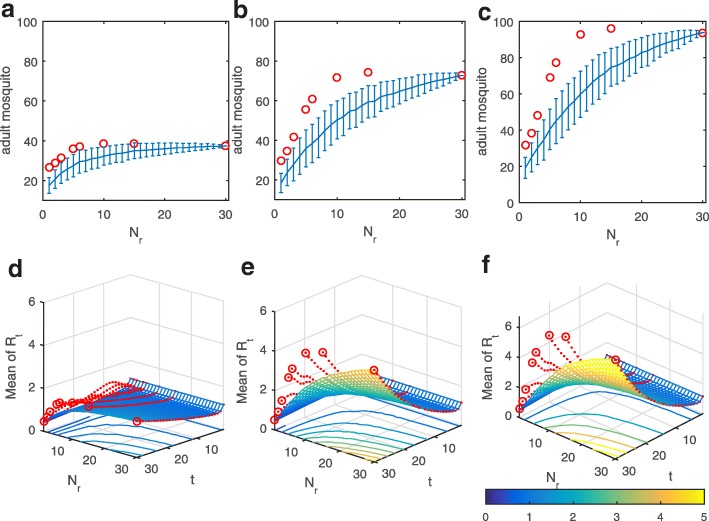
**a**-**c** The total amount of mosquitoes produced within 1 month versus the number of rainy days (*Nr* ). The blue line shows the mean and the bars show standard deviation when the precipitation data are generated randomly. The red circles show the total amount of mosquitoes when the rain is distributed regularly. **d**-**f** The effective reproduction number versus time and *Nr*. The surfaces show the mean results of 500 simulations when the precipitation data are generated randomly. The red dashed lines represent the results when the rain is distributed regularly

To investigate the effects of various distributions of precipitation on the growth of the mosquito population, we fixed the total amount of precipitation to be 100, 200 and 300 mm within one month and varied the number of rainy days from 1 to 30 (randomly distributed within a month, blue curves). The precipitation data were generated by sampling without replacement and 500 simulations were run. The mean and standard deviation of the adult mosquito population (Fig. 4a-c), show that the mean number of mosquitoes increases with an increasing number of rainy days, indicating that the more the rainy days (or the more precipitation), the larger the mosquito population. Note that the standard deviation is quite large when the number of rainy days is around 15, meaning that the risk of getting more mosquitoes is high at this point. We further investigated the amount of mosquitoes when the rainy days are regularly distributed instead of randomly generated (Fig. 4a-c, red circles), showing that the amount of mosquitoes is relatively large when the number of rainy days is greater than 10, indicating that the pattern of rainfall of every additional day contributes to increasing the mosquito population. Comparing the two (regular or random) patterns of rainfall for a given total amount of precipitation implies that a regular pattern of rainfall was more beneficial for mosquito population growth.

Repeating the above plotting allows us to examine the effect of patterns of rainfall on the mean ERN versus time (Fig. 4d-f). It follows that the mean of *R*_*t*_ also increases with an increasing number of rainy days (the blue surface in Fig. 4d-f), agreeing with the varying trend of the adult mosquito population (Fig. 4a-c). Similarly, the value of *R*_*t*_ for a regular distribution of rainfall is quite large when the number of rainy days is about 10–15, which indicates that regular rainfall, especially every other day, results in more new dengue infections, in line with the conclusion on the mosquito population development (Fig. 4a-c). Thus, for a given total amount of precipitation, different patterns of rainfall significantly affect the mosquito population and hence the occurrences of new infections of dengue fever (Fig. 4). In particular, frequent and random rainfall or regular rainfall every other day is most beneficial for the mosquito population and hence new dengue infections.

### Effects of yearly patterns of rainfall on the ERN

**Fig. 5:**
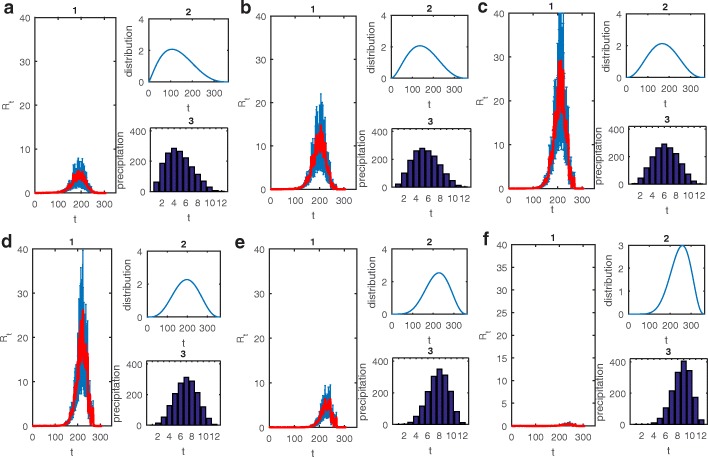
(**1**) Effective reproduction numbers under different precipitation distributions. The red line shows the mean of 150 simulations. Blue bars show the standard deviation. (**2**) The distribution used to generate precipitation data. (**3**) The monthly precipitation obtained by the generated data. **a** Using data from the distribution Be(2.24,4); **b** using Be(2.8,4); **c** using Be(3.54,4); **d** using Be(4.55,4); **e** using Be(6,4); **f** using Be(8.29,4)

**Fig. 6:**
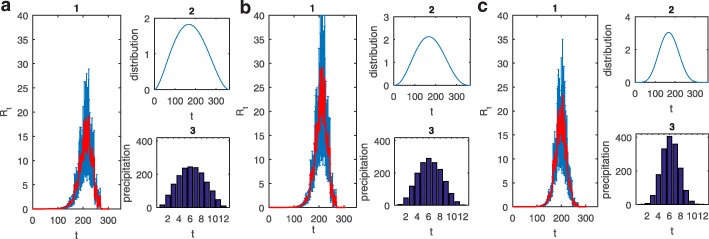
(**1**) Effective reproduction numbers under different precipitation distributions. The red line shows the mean of the 150 simulations. Blue bars show the standard deviation. (**2**) The distribution used to generate precipitation data. (**3**) The monthly precipitation obtained by the generated data. **a** Using data from the distribution Be(2.69,3); **b** using Be(3.54,4); **c** using Be(6.92,8)

To investigate the effect of various yearly distributions of precipitation on the ERN, we simulated events from March to December and plotted the ERN by using the total precipitation and the mean temperature data calculated as the mean from 2012 to 2016. Figures 5 (1) and 6 (1) show the mean and variance of the 150 simulations for the ERN, and Fig. 5 (2, 3) and Fig. 6 (2, 3) give the distributions and the monthly mean precipitation of the randomly generated data. Figures 5a-f show that as the peak of precipitation varies from Spring to Autumn, the ERN increases and then decreases. In particular, the ERNs shown in Fig. 5c, d, corresponding to the peak of precipitation reached in June and July, are larger than those in other sub-plots, which indicates that high precipitation in summer causes the risk of transmission of dengue to be more likely than high precipitation in Spring or Autumn. To further examine the effect of the degree of aggregation of rainfall on the ERN, we simulated on the basis of data generated from a distribution with the same mode (June) but different kurtosis, as shown in Fig. 6. As the kurtosis increases, the ERN increases initially and then decreases (Fig. 6a-c), indicating that an appropriate kurtosis (Fig. 6b) induces transmission to be more likely than a particularly large or particularly small kurtosis does. This indicates that high precipitation in summer together with an appropriate degree of aggregation is beneficial to the transmission.

To discuss the reliability of conclusions based on the generated data, we further investigated the correlation between the real data and the generated data (Fig. 5). The monthly precipitation data of 2012, 2013, 2014, 2015 and 2016 were significantly correlated (*P <*0*.*05) with the data generated from Beta distributions Be(2.8,4) (Fig. 5b), Be(3.54, 4) (Fig. 5c) and Be(4.55,4) (Fig. 5d) and they were most highly correlated with the generated data from distribution Be(3.54,4) (Fig. 5c) (Here, *Be*(*a, b*) is a Beta distribution with cumulative distribution function $F\left(x|a,b\right)=\frac{1}{B\left(a,b\right)}{\int}_0^x{t}^{a-1}{\left(1-t\right)}^{b-1} dt,B\left(\bullet \right)$ is the Beta function). The correlation coefficients (*P-*values) are 0.7869 (0.0024), 0.8891 (0.0001), 0.8359 (0.0007), 0.7592 (0.0042) and 0.8264 (0.0009), respectively. These results indicate that the yearly pattern of precipitation with a peak in summer (Fig. 5b-d) is more consistent with the actual rainfall in Guangzhou. Moreover, we investigated the correlation between the real data and the generated data (Fig. 6) to further identify the yearly patterns of rainfall that significantly affect the ERNs. Additional file 1: Table S4 shows that the precipitation data in 2013 are the most strongly correlated with the generated data (Fig. 6b), which means that the pattern of the yearly distribution of rainfall that occurred in 2013 is the most likely to cause the transmission of dengue, with the precipitation in 2014 and 2016 being the second and third most strongly correlated with the generated data (Fig. 6b). The precipitation data in 2015 were most strongly correlated with the generated data with the smallest ERN (Fig. 6c), indicating that the precipitation distribution in 2015 was the most disadvantageous to the transmission.

We ranked the factors that contribute to the ERN in Table 2, which provides insights and detailed reasons for the outbreak level for each year, for details of which and methods used see Additional file 1: Text S1.

**Table 2:** Rank list of those factors that influenced the effective reproduction number in different years

	1st	2nd	3rd	4th	5th
Temperature	2014	2015	2012	2013	2016
Total precipitation	2016 (2117.3)^a^	2015 (1856.8)	2014 (1629.4)	2013 (1535.6)	2012 (1131.8)
Total rainy days	2016 (162)^b^	2015 (140)	2014 (137)	2013 (130)	2012 (111)
Yearly pattern of rainfall	2013	2014	2016	2012	2015

^a^Total precipitation

^b^Total rainy days

## Discussion

Recently, dengue fever has shown a persistently increasing trend and multi-year oscillations and has become a public health concern in southern China. The 2014 outbreak in Guangdong Province was the most serious one in mainland China to date. Thus, identification of the key factors which contributed to it and an ability to make early predictions of high levels of dengue transmission within a season are urgent requirements for improving control strategies. Here we proposed a hybrid model incorporating a stage-structured dynamic model for the mosquito population and a Poisson model for dengue transmission. We focused on analyzing the effects of temperature, precipitation and various patterns of rainfall on the ERN, after fitting a model to the BI data of 2014 and 2015. We then validated the model on the basis of the 2016 and 2017 BI data. We identified what kind of rainfall pattern is conducive to transmission and provided explanations of why outbreaks can be big or small.

The BI data, calculated according to the number of containers with mosquito eggs or larvae per 100 houses, can be affected by the time and place of data acquisition and control measures. Besides, the weather conditions in the diapause period of the mosquitoes may affect the size of the mosquito populations. However, the minor discrepancies between our results and the data are acceptable despite these stochastic factors. On the basis of our parameter values, we obtained the ERNs from 2012 to 2017 (Fig. 1d), which agree with the case data (Fig. 1c). Fig. 1d shows that the 2014 ERN peak was the largest with a value of almost 5, between the peak values of the ERNs of Haizhou and Zengcheng districts [26]. This is reasonable as the ERN of the entire Guangzhou city often lies between the ERNs of areas with either serious or minor outbreaks.

By keeping the uniform precipitation each year as the mean of the precipitation from 2012 to 2016, we found that the temperature in 2014 (or 2016) was the most suitable (unsuitable) for transmission (Fig. 1f). In addition, by keeping the uniform temperature each year as the mean temperature from 2012 to 2016 the results showed that the precipitation pattern that occurred in 2016 provided the highest risk factor for inducing a major outbreak. It follows from Fig. 2 that increasing the precipitation leads to an increase in ERNs, but with lags of more than 10 days, when the temperature is between 25–30 °C. This delay corresponds with one or two weeks between oviposition and adult mosquito emergence and there is also a latent period in the dengue transmission process. In contrast, the effect of temperature on the ERNs was observed immediately since it not only determines the development rates and life spans of themosquitoes but it is also embodied in the transmission process.

Main foci of this paper were examinations of the effects of different patterns of precipitation, including monthly and yearly patterns, on dengue transmission. Figure 3 provides strong evidence that increasing precipitation leads to an increase in the mosquito population size with different distributions of rainy days causing very different abundances of mosquitoes. Moreover, Fig. 4 reveals that uninterrupted drizzle is beneficial to the reproduction of mosquitoes, and hence is highly conducive to the transmission. This was verified in Fig. 1f, in which the mean precipitation data from 2012 to 2016 were employed to yield uninterrupted drizzle, giving ERN values for every year much larger than in any of the other scenarios (Fig. 1d). Besides, comparing Fig. 1e and f, it seems that the variation in temperature rather than in rainfall is more important to determine the variations in ERN. In fact, this difference may be due to the average of the precipitation data yielding uninterrupted drizzle.

For the yearly distribution of precipitation, we found that the distribution with a peak in June and July with marked kurtosis induced a relatively large ERN, which is the most propitious for the transmission of dengue (Figs. 5c-d, 6). Further, taking the delay of the effects of precipitation into consideration (Fig. 3), we concluded that a rainfall peak in late June and possibly to August can lead to much larger ERNs than peaks in any other months can. Indeed the peak of the monthly mean temperature in Guangzhou is from June to August. Hence, our results reveal that large amounts of rain in a season of high temperature are beneficial for dengue transmission. In fact, the annual cycle of rainfall in Guangzhou indeed peaks in June and July. So, our results also confirm why dengue fever is prevalent in Guangzhou. In particular, much rain and high temperatures in Guangzhou during June to August and a suitable monthly distribution of precipitation (i.e. frequent and random rain or regular rain every two days; Fig. 4) will lead to relatively large ERNs and hence to more new dengue infections. These results provide a strong basis for determining a season that has a large outbreak.

In summary, we quantified and evaluated the impact of climate factors on dengue fever. Our findings reveal danger signals of climate leading to the induction of a major dengue fever outbreak. These signals are relatively high temperature, high total precipitation with a rainfall peak in June and July, together with uninterrupted drizzle or regular rainfall every two days. Taking into account delays between rainfall and dengue transmission, we note that the peak of dengue incidence usually appears in September in Guangzhou. Therefore, current precipitation patterns and near-future predictions of temperature and precipitation variation permit more effective prevention and control measures for annual outbreaks, thereby permitting rapid response management decisions. Thus, our analyses can provide a theoretical basis for early warning of possible within-year major outbreaks to inform public health planning and rapid responses to mosquito-borne outbreaks. Additionally, these weather conditions favourable to dengue transmission may also be key factors for inter-annual variations of dengue fever. However, predicting multi-year cycles based on our current data is very difficult, which depends on multiple factors including the evolution of the virus, the immune level of the population, the susceptibility of the population and so on, topics for future research.

## Conclusions

In the present study, a hybrid model was proposed to analyze the effects of temperature, precipitation and various patterns of rainfall on the ERN of dengue based on data from Guangzhou, China. The findings show a set of weather conditions favourable to a future major dengue outbreak. Such information permits seasonal rapid response management decisions due to the lags between the precipitation events and the realisation of the ERN.

## Additional file

Additional file 1: **Text S1.** Data and methods. **Table S1.**Total and imported cases from 2012 to 2017. **Table S2.** Definitions of the parameters used in the model. **Table S3.** Results of the MCMC algorithm. **Table S4.** Correlation coefficients (*P*-values) between the precipitation data and the data generated from the Beta distributions. (DOCX 101 kb)

## Abbreviations

BI: Breteau index
ERN: Effective reproduction number
EIP: Extrinsic incubation period
MCMC: Markov Chain Monte Carlo
DTR: Daily temperature range
