# Supplementary material for: A combination of climatic conditions determines major within-season dengue outbreaks in Guangdong Province, China
Source: Parasit Vectors. 2019 Jan 21;12:45. doi: 10.1186/s13071-019-3295-0 (PMC6341621; doi:10.1186/s13071-019-3295-0)
Supplement: Supplementary file 1 — Text S1. Data and methods. Table S1.Total and imported cases from 2012 to 2017. Table S2. Definitions of the parameters used in the model. Table S3. Results of the MCMC algorithm. Table S4. Correlation coefficients (P-values) between the precipitation data and the data generated from the Beta distributions. (DOCX 101 kb) [file 13071_2019_3295_MOESM1_ESM.docx]

Additional file 1

A combination of climatic conditions determines major within-season dengue outbreaks in Guangdong Province, China

**Text S1 Data and Methods**

Weather and Case Data Acquisition

The Guangzhou temperature and precipitation data from 2012 to 2017 were obtained from (http://weather.org/weatherorg_records_and_averages.htm) for calculating the daily mean temperature and the daily temperature range (DTR). We then estimated the temperature at any time within a day using the sinusoidal hourly temperature variation between the maximum and minimum [1]. We obtained dengue cases data from the Guangdong provincial Health and Family Planning Commission [(http://www.](http://www/)gdwst.gov.cn/; TableS1 and Fig.1a,b. From 1990 to 2014 there were 69321 cases with 11 deaths in mainland China [2], where the biggest outbreak was in 2014 with a total of 47056 cases, 45230 of which were in Guangdong province. The daily numbers of new cases in Guangzhou city during September 22nd to October 30th, 2014 are shown in Fig.1a.

Climate dependent parameters

As temperature has a major effect on insect development, the egg-laying, development and mortality rates are assumed to be temperature dependent following the explicit expressions:

$$\begin{matrix} b(t)=b_{0}+\frac{E_{\max}}{1+\exp\left(-\frac{W(t)-E_{mean}}{E_{\mathrm{var}}}\right)}; \\ \mu_{i}(t)=1-\mu_{0i}\exp\left(-\frac{T(t)-T_{0}}{\nu}\right)^{2}; \\ d(t)=A\frac{T(t)+K}{298.15}\frac{\exp\left(\frac{HA}{1.987}\left(\frac{1}{298.15}-\frac{1}{T(t)+K}\right)\right)}{1+\exp\left(\frac{HH}{1.987}\left(\frac{1}{TH}-\frac{1}{T(t)+K}\right)\right)}, \end{matrix}$$

where $b_{0}$is the baseline egg laying rate, $E_{\max}$is the maximum egg laying rate above the baseline, $E_{mean}$is the value at which the moisture index produces 50% of$E_{\max}$,$E_{\mathrm{var}}$is the variance;$\mu_{0i}(i=1,2)$is the baseline survival rate,$T(t)$is the temperature at time$t$with$T_{0}$being the optimal temperature for survival,$v$is the variance. We adopt the formula of the development rate$d(t)$for *Aedes* here. This is the Sharpe & DeMichele equation [3] parameterized for *Culex* with laboratory data by [4].$A$is the development rate assuming no temperature in activation of an enzyme critical for development,$HA$is the enthalpy of activation of the reaction that is catalyzed by the enzyme (cal *mol^−^*1), $HH$is the enthalpy change associated with high temperature inactivation of the enzyme(cal *mol^−^*1), $K$is the air temperature in Kelvin units, $TH$is the temperature where 50% of the enzyme is in activated by high temperature.

Temperature-dependent rates involved in *Rvc* were obtained from published studies [1,5,6]:

$$\begin{matrix} a(t)=a_{1}T(t)+a_{2},12.4\leq T(t)\leq32; \\ n(t)=a_{3}+\exp(a_{4}-a_{5}T(t)); \\ b_{m}(t)=\left\{\begin{matrix} a_{6}T(t)-a_{7},12.4\leq T(t)\leq26.1; \\ 1,\begin{matrix} & & \begin{matrix} & 26.1<T(t)\leq32.5 \end{matrix}; \end{matrix} \end{matrix}\right. \\ b_{h}(t)=a_{8}T(t)(T(t)-12.286)\sqrt{32.461-T(t)},12.286\leq T(t)\leq32.461. \end{matrix}$$

Let$i_{t}$be the number of daily newly infected cases at day$t$. Let$p_{j}$,where$j$is the infection age, be the relative frequency (generation time) of secondary transmission with respect to the time since infection of a primary case. Then the total infectivity of infected individuals $\Lambda_{t}$at time$t$is:

$$\Lambda_{t}=\sum_{j=1}^{\min(t,k)} p_{j}i_{t-j},$$

where$k$is the maximum value of the serial interval. The mean number of local incident cases at time $t$is given by the product of total infectivity and the instantaneous reproduction number

$$\varphi_{t}=\Lambda_{t}R(t)=R(t)\sum_{j=1}^{\min(t,k)} p_{j}i_{t-j}.$$

Hence, we postulate an epidemic process.

Parameter estimation

Parameters involved in the mosquito dynamics model are derived from a previous paper [7] (see TableS1), in which parameters were estimated using the BI data of 2014 and 2015. Initial values of the mosquito dynamics model (1) are assumed to be (2*,*0) [7] unless there is a special description. The infectious period $T_{h}$ is assumed to be 5 (days), and the maximum value of the serial interval can also be obtained according to the previous paper [8,9]. Other unknown parameters in the transmission model were estimated by the Markov Chain Monte Carlo (MCMC) method and the generation interval-informed method [10](see Table S2). To estimate these unknown parameters, we assumed that the number of local incident cases$i_{t}$follows a Poisson distribution with mean $\varphi_{t}$, i.e., $i_{t}\sim Pois(\varphi_{t})$*,* so the likelihood function (denoted by *LF* ) is given by

$$LF=\prod_{t=1}^{39} \frac{\exp(-\varphi_{t})\varphi_{t}^{i_{t}}}{\Gamma(i_{t}+1)},\varphi_{t}=\Lambda_{t}R_{t}=R_{t}\sum_{j=1}^{\min(t,k)} p_{j}i_{t-j}.$$

where$\Gamma(\cdot)$is the gamma function, and $p_{j}$is assumed to follow a gamma distribution with mean 14 and variance 2 [9]. Anadaptive Metropolis-Hastings (M-H) algorithm was carried out for the MCMC procedure, and after a burn-in period of 50000 iterations the next 10000 samplers provided the estimates. The prior distributions were chosen to be a no information prior distribution, namely, the uniform distribution. The domain of every parameter was chosen according to previous studies [6,9]. Results of the MCMC algorithm including the mean, standard variance and geweke test results are shown in TableS2.

Model validation

Parameter values of the mosquito dynamics model were estimated by fitting the immature stage of mosquitoes and the BI data in 2014 and 2015 [7]. We used the BI data in 2016 and 2017 to conduct the model validation. The fitting and simulation results are shown in Fig.1c, which shows the strong consistency between the simulation and the BI data in 2016, while the simulation result is a little higher than the BI surveillance data in 2017. This may have been caused by stochastic factors or control measures. It follows that the simulated number of immature mosquitoes in 2017 is higher than that in 2016, which is in agreement with the variation trend of the data. The simulation results agree well with the data for 2015 and 2016, validating the model’s rationale and the parameter estimations.

Simulation methods

Methods used to do simulations in the main text are shown as follows:

Fig.1(c): Due to the low temperatures and short sunshine periods in winter, we conducted simulations only between March and November each year. The initial value of the immature stage in every year is chosen to be the ﬁrst BI data in March of 2015. The initial value for adult mosquitoes and the moisture index are assumed to be zero.

Fig. 3: We fixed the value of the daily mean temperature to be 30 and DTR to be 5 since the most suitable season for the spread of dengue fever is summer. The initial value of the mosquito model is chosen to be (2*,*2). Let the total precipitation vary from 1 to 200 and the number of rainy days be fixed at 10 with equal rainfall each time. We used sampling without replacement to generate the date of rainfall from 1st to 30th of a month, and plotted the distributions in Fig.3a, in which every row shows a time distribution of precipitation with the dark blue representing the day without rain and the red the rainy day.

Fig.5 and Fig.6: The date of rainfall was generated by replacement sampling from Beta distributions with parameters listed in Table 3 and then was mapped to the interval [1*,*365]. Suppose the rainfall was equal each time. The total precipitation was fixed to be the mean precipitation from 2012 to 2016 and the sample size was assumed to be the mean number of rainy days from 2012 to 2016. To remove the effect of randomness, 150 groups of generated data were generated from one distribution and the mean and variance of the 150 simulations for the effective reproduction number are given in Fig. 5(1) and Fig.6(1). The distribution and the monthly mean precipitation of the randomly generated data are shown in Fig. 5(2-3) and Fig. 6(2-3).

Ranking procedure

For the total precipitation and total number of rainy days, we ranked them according to the real data from the largest to the smallest. The temperatures in 2014, 2015 were ranked first and second for inducing high ERNs (Fig.1f). For the yearly pattern of rainfall, Table S3 shows that the pattern of precipitation data in 2013, the most highly correlated with the generated data (Fig. 6b), was the most likely factor to cause high transmission rates, although the total precipitation and total number of rainy days were highest in 2016, when the temperature ranked last regarding its contribution to the ERN (Table 2). The precipitation distribution in 2013 was the most suitable for causing a high ERN, while the temperature, total precipitation and total number of rainy days in 2013 ranked second to last. Hence large outbreaks did not occur in 2013 or 2016. Interestingly, the temperature in 2014 was the most suitable for causing a high ERN and hence a high transmission rate. The precipitation distribution, the total precipitation and the total number of rainy days ranked the second and third best contributors to the ERNs. These integrated factors and their combination induced the major outbreak in2014.

Tables

Table S1: Total and imported cases from 2012 to 2017.

| year 2012 | 2013 | 2014 | 2015 | 2016 | 2017 |
| --- | --- | --- | --- | --- | --- |
| Total cases 154 [11] | 1332[11] | 373401 | 992 | 1773(10.23) | 793(8.23) |
| Imported cases 15 | 83 |  | 45 |  | 36 |

- - 1. *chttp*:[*//w*](http://www.gzcdc.org.cn/News/V)*ww*[*.gzcdc.org.cn/News/V*](http://www.gzcdc.org.cn/News/V)*iew.aspx*?*id*=1904;
    2. *http* : *//news.timedg.com/*2016 *−*01*/*17*/*20338950*.shtml*;
    3. *http*:[*//w*](http://www.gdwst.gov.cn/)*ww*[*.gdwst.gov.cn/*.](http://www.gdwst.gov.cn/)

Table S2: Definitions of the parameters used in the model

| Parameter | Definition(Units) | Value | References |
| --- | --- | --- | --- |
| $b_{0}$ | the baseline egg laying rate | 2.4337 | 7 |
| $E_{\max}$ | the maximum egg laying rate above baseline | 2.9147 | 7 |
| $E_{mean}$ | the value at which the moisture index produces 50% of Emax | 0.0024 | 7 |
| $E_{\mathrm{var}}$ | the variance | 4.0471 | 7 |
| $A$ | the development rate assuming no temperature inactivation of the critical enzyme | 0.1508 | 7 |
| $HA$ | the enthalpy of activation of the reaction that is catalyzed by the enzyme (cal *mol^−^*1) | 39949.6 | 7 |
| $HH$ | the enthalpy change associated with high | 28007.4 | 7 |
| $K$ | the air temperature in Kelvin units | 273.15 | 4 |
| $TH$ | the temperature where 50% of the enzyme is inactivated by high temperature | 298.8704 | 7 |
| $\mu_{01}$ | the baseline survival rate of immatures | 0.9514 | 7 |
| $\mu_{02}$ | the baseline survival rate of adults | 0.5943 | 7 |
| $T_{01}$ | the optimal temperature for survival of the immature | 16.0427 | 7 |
| $\nu_{1}$ | the variance for immatures | 6.2841 | 7 |
| $T_{02}$ | the optimal temperature for survival of the adults | 21.0372 | 7 |
| $\nu_{2}$ | the variance for the adults | 13.4776 | 7 |
| $\delta$ | Evaporation rate | 0.6094 | 7 |

Table S3: Results of the MCMC algorithm.

|  | mean | std | *MCerr* | tau | geweke |
| --- | --- | --- | --- | --- | --- |
| *c/Nh* | 5.7621 | 2.4118 | 0.14307 | 38.557 | 0.97758 |
| *a*1 | 0.016045 | 0.0017109 | 0.0001189 | 35.643 | 0.98439 |
| *a*2 | 0.069927 | 0.0116 | 0.00085665 | 41.621 | 0.91554 |
| *a*3 | 3.6499 | 0.14362 | 0.009325 | 30.414 | 0.9819 |
| *a*4 | 4.8494 | 0.34425 | 0.024172 | 36.836 | 0.98539 |
| *a*5 | 0.16352 | 0.016408 | 0.00094288 | 31.613 | 0.9658 |
| *a*6 | 0.060611 | 0.0057082 | 0.00029316 | 37.612 | 0.95442 |
| *a*7 | 0.59654 | 0.058383 | 0.0037129 | 33.208 | 0.96456 |
| *a*8 | 0.0014409 | 0.00031471 | 1.6656e-05 | 43.837 | 0.90031 |

TableS4: Correlation coefficients (p values) between the precipitation data and the data generated from the Beta distributions.

|  | 2012 | 2013 | 2014 | 2015 | 2016 |
| --- | --- | --- | --- | --- | --- |
| Be(2.69,3)-Fig.6(A) | 0.7300(0.0070) | 0.8765(0.0002) | 0.8209(0.0011) | 0.7302(0.0070) | 0.7831(0.0026) |
| Be(3.54,4)-Fig.6(B) | 0.7869(0.0024) | 0.8891(0.0001) | 0.8359(0.0007) | 0.7592(0.0042) | 0.8264(0.0009) |
| Be(6.92,8)-Fig.6(C) | 0.8023(0.0019) | 0.7980(0.0019) | 0.7953(0.0020) | 0.8108(0.0014) | 0.8232(0.0010) |

References

1. Lambrechts L, Paaijmans KP, Fansiri T, Carrington LB, Kramer LD, Tomas MB, et al. Impact of daily temperature ﬂuctuations on dengue virus transmission by *Aedes aegypti*. Proc Natl Acad Sci USA.2011; 108:7460-7465.

2. Li C, Wang X, Wu X, Liu J, Ji D, Du J. Modeling and projection of dengue fever cases in Guangzhou based on variation of weather factors. Sci Total Environ. 2017; 605-606:867-873.

3. Rueda LM, Patel KJ, Axtell RC, Stinner RE. Temperature dependent development and survival rates of *Culex quinquefasciatus* and *Aedes aegypti* (Diptera:Culicidae). J Med Entomol. 1990; 27:892-898.

4. Gong H, DeGaetano AT, Harrington LC. Climate-based models for West Nile *Culex* mosquito vectors in the Northeastern US. Int J Biometeorol. 2011; 55: 435-446.

5. Brady OJ, Golding N, Pigott DM, Kraemer MUG, Messina JP, Reiner J, et al. Global temperature constraints on *Aedes aegypti* and *Ae.albopictus* persistence and competence for dengue virus transmission. Parasit Vectors. 2014; 7: 338.

6. Liu-Helmersson J, Stenlund H, Wider-Smith A, Rocklov J. Vectorial capacity of *Aedes aegypti*: eﬀects of temperature and implications for global dengue epidemic potential. PLoS One. 2014; 9:e89783.

7. Wang X, Tang S, Cheke RA. A stage structured mosquito model incorporating effects of precipitation and daily temperature fluctuations. J Theor Biol. 2016; 411:27-36.

8. World Health Organization (WHO), 2015. Dengue and severe dengue-Factsheet. Available: <http://www.who.int/mediacentre/factsheets/fs117/en/.>

9. Jing Y, Wang X, Tang S. Data informed analysis of 2014 dengue fever outbreak in Guangzhou: Impact ofmultiple environmental factors and vector control. J Theor Biol. 2017; 416:161-179.

10. White LF, Pagano M. A likelihood-based method for real-time estimation of the serial interval and reproductive number of an epidemic. Stat Med. 2008; **27**:2999-3016.

11. Cao Q, Luo L, Jing QL, Li YL, Wei YH. Epidemiological characteristics of dengue fever in Guangzhou City(2012-2013). Strait J PrevMed. 2014; 20:1007-2705(2014)06-0001-03.
